# High Resolution Ambient MS Imaging of Biological Samples by Desorption Electro-Flow Focussing Ionization

**DOI:** 10.1021/acs.analchem.2c00345

**Published:** 2022-07-05

**Authors:** Vincen Wu, Jocelyn Tillner, Emrys Jones, James S. McKenzie, Dipa Gurung, Anna Mroz, Liam Poynter, Daniel Simon, Cristina Grau, Xavier Altafaj, Marc-Emmanuel Dumas, Ian Gilmore, Josephine Bunch, Zoltan Takats

**Affiliations:** † Department of Digestion, Metabolism and Reproduction, Imperial College London, Sir Alexander Fleming Building, London SW7 2AZ, United Kingdom; ‡ Department of Surgery & Cancer, Metabolism and Reproduction, Imperial College London, Sir Alexander Fleming Building, London SW7 2AZ, Unite Kingdom; § NiCE-MSI, National Physical Laboratory (NPL), Hampton Road, Teddington, Middlesex TW11 0LW, United Kingdom; ∥ Waters Corporation, Altrincham Road, Wilmslow SK9 4AX, United Kingdom; ⊥ Biological Mass Spectrometry, Rosalind Franklin Institute, Harwell Campus, Didcot OX11 0QS, United Kingdom; # Neurometabolic Unit, Department of Neurology, Hospital Sant Joan de Déu, 08950 Barcelona, Spain; ¶ Neurophysiology Laboratory, Department of Biomedicine, Faculty of Medicine and Health Sciences, Institute of Neurosciences, University of Barcelona, Barcelona 08036, Spain

## Abstract

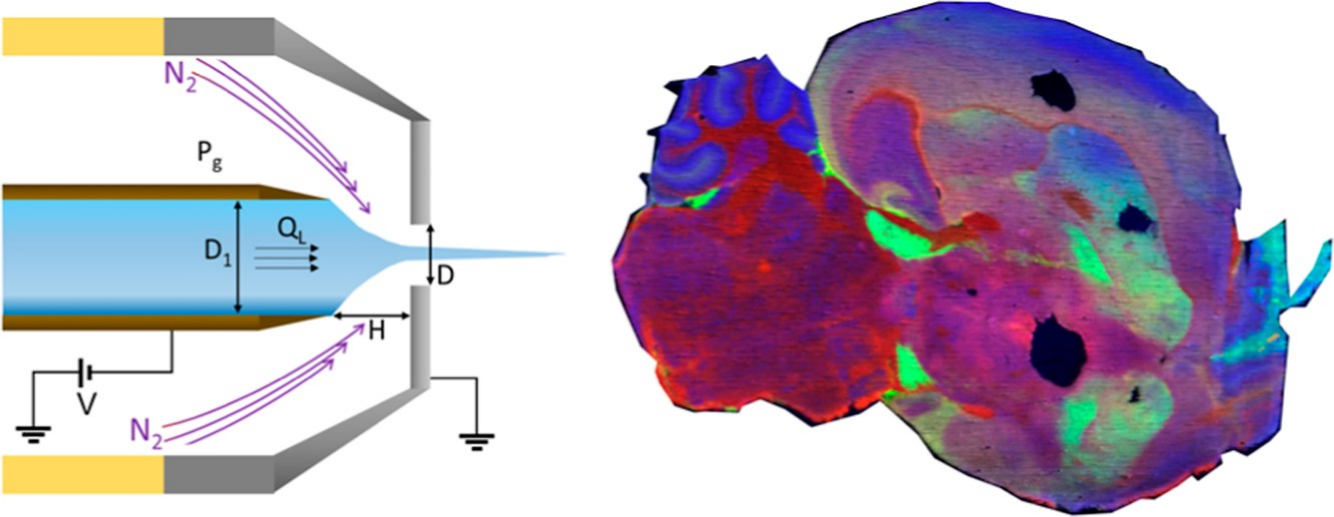

In this study, we examine the suitability of desorption electro-flow focusing ionization (DEFFI) for mass spectrometry imaging (MSI) of biological tissue. We also compare the performance of desorption electrospray ionization (DESI) with and without the flow focusing setup. The main potential advantages of applying the flow focusing mechanism in DESI is its rotationally symmetric electrospray jet, higher intensity, more controllable parameters, and better portability due to the robustness of the sprayer. The parameters for DEFFI have therefore been thoroughly optimized, primarily for spatial resolution but also for intensity. Once the parameters have been optimized, DEFFI produces similar images to the existing DESI. MS images for mouse brain samples, acquired at a nominal pixel size of 50 μm, are comparable for both DESI setups, albeit the new sprayer design yields better sensitivity. Furthermore, the two methods are compared with regard to spectral intensity as well as the area of the desorbed crater on rhodamine-coated slides. Overall, the implementation of a flow focusing mechanism in DESI is shown to be highly suitable for imaging biological tissue and has potential to overcome some of the shortcomings experienced with the current geometrical design of DESI.

## Introduction

Since their inception in the early 2000s, ambient ionization techniques have become increasingly popular with a multitude of applications, including forensics,^1^ pharmaceutical analysis,^2^ and food analysis,^3^ due to their ease of use and requirement for only minimal sample preparation. Desorption electrospray ionization (DESI) is one of the first and most commonly used ambient ionization techniques, particularly with regard to mass spectrometry imaging (MSI) of biological tissue.^4−6^ It allows spatial mapping in tissue of endogenous molecules, such as metabolites,^7^ lipids,^8^ peptides and proteins,^9^ and also drugs and their metabolites.^10^ In some cases, DESI is able to ionize molecules that are not amenable by other methods, such as MALDI.^10^ However, like many other ambient ionization techniques, DESI suffers from repeatability issues and the performance is very much dependent on the setup used and on user experience.^11^ The key component within the system is the sprayer.^12^ It has been shown that the scan direction in DESI-MS imaging (DESI-MSI) has an impact on signal intensity,^13^ which is attributed to sprayer asymmetry.^14^ Therefore, a suboptimal sprayer positioning or orientation can have a deleterious effect on the signal. The DESI sprayer classically consists of a solvent emitter (usually fused silica) surrounded by a second capillary or metal cone that delivers a nebulizing gas flow. Ideally, these two capillaries or tubes are concentric; in practice, they rarely are, leading to variability in the primary electrospray and signal intensity.^14^

**Figure 1:**
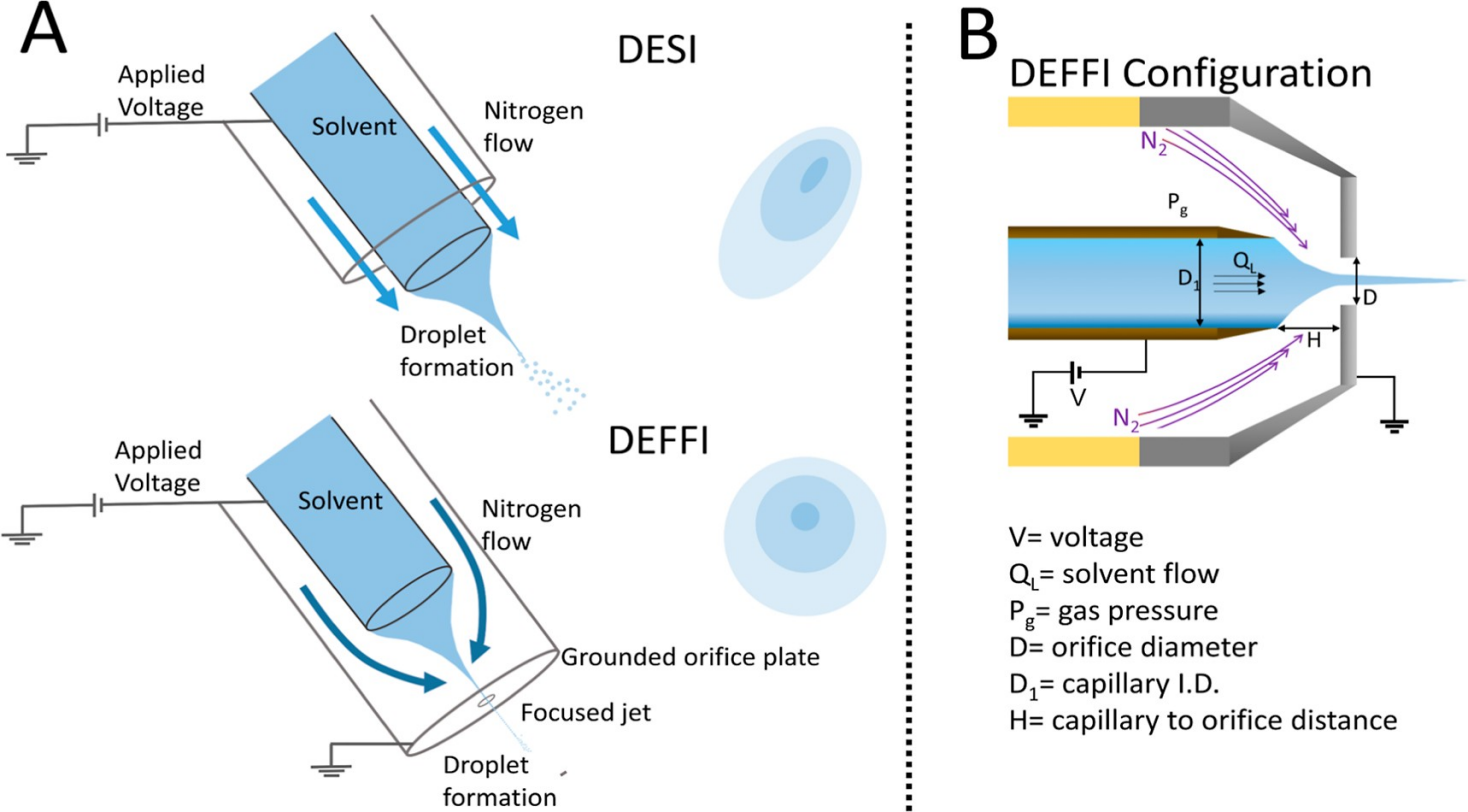
DEFFI and DESI setups and DEFFI parameters. (A) DEFFI and DESI sprayer setups (not to scale) and corresponding desorption footprints. The impact site of DESI spray is often elliptical due to asymmetry of the sprayer, while the DEFFI spray is rotationally symmetric and distorted only by the angle of incidence of the sprayer with the surface. (B) Illustration of the DEFFI configuration is shown with the corresponding parameters.

Thomas Forbes et al.^15^ have reported an approach termed DEFFI, in which an electro-flow focusing nebulizer^16^ is used. In this configuration, the solvent delivery capillary is retracted inside the sprayer behind the orifice in the nozzle (see Figure 1A). This alternative approach requires lower gas pressures (10–20 psi) and lower voltages (0.5–2 kV) than those previously reported for the conventional DESI approach, in keeping with the underlying mechanistic differences. The resultant solvent spray at comparable flow rates is more tightly focused and rotationally symmetrical than the DESI approach, which would suggest a greater suitability for surface mapping approaches.

The basic principle of flow focusing is that the shear forces exerted on a flowing liquid by a co-flowing gas become so large that they overcome surface tension, preventing droplet formation at the orifice and leading to the formation of a steady jet.^16^ This jet is much smaller in diameter than the inner diameter of the solvent capillary or the orifice and breaks up into steady microjets of micron-sized, mono-disperse droplets further downstream. If the shear forces are insufficient to overcome surface tension, dripping rather than jetting occurs, whereby large droplets of the liquid are detached from the end of the solvent capillary by the gas.^17^ In jetting, the size, but also the stability of the jet, depends primarily on the viscosity and flow rate of the liquid and the pressure drop of the co-flowing gas.^16^ This means that the size of the jet can be reduced by reducing the liquid flow rate, though not indefinitely. Gañán-Calvo et al.^18^ found that by applying a voltage between the feed capillary and the orifice plate, the jet could be charged, further reducing the droplet size by a factor of up to 10 in a process termed “electro-flow focussing”. In addition, the orifice plate is grounded, which means that the potential is not applied between the sprayer and the mass spectrometer inlet but is self-contained. As the orifice is only hundreds of micrometers away from the solvent emitter, the same charge densities present in DESI can be achieved by applying a much smaller voltage as well as reducing space charge effects. A detailed mechanistic explanation of coupling electrospray and flow focusing techniques was described by Gañán-Calvo et al.^18^

DESI-MSI is increasingly being used for imaging clinical tissue specimens with a view to augmenting classical histopathology, whereby tissue types can be classified according to their mass spectral profiles using multivariate statistical tools.^19−21^ Currently, DEFFI MSI has been applied only to artificial fingerprints where the localization of fatty acids and trace exogenous compounds, such as explosives, narcotics, and lotion, was mapped.^22^ The flow focusing mechanism has not yet been tested on biological tissue samples. It is envisaged that such an approach would offer performance enhancements for biological tissue imaging in the way of improved spatial resolution and signal intensity but equally importantly in the robustness of analysis, providing an easier and more reproducible experimental setup. This will be significant if data from multiple instruments and multiple laboratories are to be compared in large studies.

In this investigation, we will implement the flow focusing mechanism into the existing commercially available DESI sprayer. The unique geometrical design of a DEFFI sprayer provides the end user with a simpler, more robust, and reliable workflow in the generation of charged droplets. It is known from studies into other ambient and surface analysis techniques that the behavior of biological tissues can be very different from that of more idealized surfaces, and therefore, the effect of the geometrical and conditional parameters of DEFFI on performance of the imaging of biological tissues must be investigated to determine the optimal conditions for this application.

It is therefore of interest to explore, examine, and optimize a wide range of the following parameters as presented in Figure 1B: (1) the shape of the distal tip of the emitter; (2) inner diameter of emitter (*D*_1_); (3) solvents; (4) gas pressures (*P*_g_); (5) distance between emitter and orifice (H); (6) orifice diameter (*D*); (7) voltage (*V*); (8) solvent flow rate (*Q*_L_); and (9) grounding of the orifice plate. In addition to improving signal intensity, we will examine how different parameters affect image resolution. Image resolution depends on the size of the primary electrospray/jet or, rather, the spread of droplets hitting the surface and any subsequent delocalization or “washing effects” from the solvent. This means that a higher spatial resolution usually comes at the price of lower intensity, as fewer molecules are sampled as the desorbed area is decreased. We examine the potential of DEFFI to be applied to imaging of biological tissues and compare its performance to DESI from the same sprayer, both in terms of signal intensity and spatial resolution.

## Experimental Section

### Materials

Methanol, ethanol, acetonitrile, isopropanol, and water ChromasolvLC–MS grade were purchased from Sigma-Aldrich (St Louis, USA) . STAEDTLER red Lumocolor pen was used to cover SuperFrost Plus Glass slides (Thermo Fisher Scientific Inc, Waltham, USA). Food grade pork liver was bought at a local supermarket (Sainsbury’s, London, UK). Mice were purchased from Charles River and bred in the Animal Facilities of the Barcelona Biomedical Research Park (PRBB, Barcelona, Spain, EU). All animal procedures met the guidelines of the European Community Directive 86/609/EEC and were approved by the Local Ethics Committee. Mice were housed under a 12:12 h light–dark schedule (lights on at 8:00 a.m.) under controlled environmental conditions of humidity (60%) and temperature (22 °C ± 2 °C) with food and water *ad libitum*. Mice were euthanized in a CO_2_ euthanasia chamber, immediately followed by brain hemispheres dissection, snap-freezing in liquid N_2_, and sample storage at −80 °C. All samples were cryosectioned at 10 μm thickness using a Microm HM 550 Cryostat (Thermo Fisher Scientific Inc., Waltham, USA). Tissue sections were thaw-mounted onto SuperFrost Plus Glass slides (Thermo Fisher Scientific Inc., Waltham, USA). Tissue sections were stored at −80 °C until further use. The tissue sections were allowed to thaw for approximately 5 min at room temperature prior to MSI. Following analysis, the tissue sections underwent hematoxylin and eosin staining followed by histopathological examination.

### Sprayer

The DEFFI sprayer was modified from the existing commercially available DESI sprayer (Waters, Wilmslow, UK) manufactured with the exact same setup according to Tillner et al*.*^23^ The sprayer consisted of a PEEK body, a stainless steel gas cone nozzle with an orifice equal to or less than 400 μm, gas fitting, and solvent fitting.^23^ A shorter and retracted solvent capillary was held by a stainless steel emitter guide, and the solvent capillary was concentrically positioned by a stainless steel disc with radially arranged holes for the flowing gas.

### Mass Spectrometry Instrumentation

Optimization and imaging experiments were performed on a Xevo G2-XS QToF (Waters, Wilmslow, UK) equipped with 2D DESI sample stage (Prosolia Inc., Indianapolis, USA). All experiments were carried out in a negative ion mode.

### Parameter Optimization

All parameters were optimized by examining the sum intensity of a selected fatty acid [FA(18:2)] and a selected phosphatidylinositol [PI(20:4/18:0)].

The optimized parameters were as follows (Figure S1): (**A**) distance between emitter and orifice (H: 0, 50,100, 200, 300, 400, 500, and 600 μm); (**B**) orifice diameter (DEFFI D: 150 and 400 μm; DESI D: 400 μm); (**C**) voltage (0, 1.5, 3, 4, 4.3, 4.5, and 5 kV); (**D**) solvent flow rate (Q_L_: 0.75, 1.5, 3, 4.5, 6, 7.5, and 9 μL/min); (**E**) gas pressures (*P*_g_: 1–10 bar); (**F**) the shape of the distal tip of the emitter: SilicaTip (New Objective, Woburn, USA), TaperTip (New Objective, Woburn, USA), and blunt capillaries; (**G**) solvents (ACN, EtOH, IPA, and MeOH), using 95:5 solvent-to-water ratio; (**H**) grounded and non-grounded orifice plate (metal cone) at 4.5 kV. While optimizing for each of the parameters, the remaining parameters were fixed at 150 μm orifice diameter, 20 μm I.D./360 μm O.D blunt tip capillary, ∼400 μm distance between emitter and orifice, 4.5 kV, 5 bar, and solvent flow rate at 1.5 μL/min with a solvent composition of 95:5 methanol–water, and the metal cap was grounded to earth.

Acquisition was performed on 5 lines of 5 mm in length on pork liver sections with 1 scan/sec at an acquisition speed of 100 μm/s and spacing between row lines of 500 μm, to prevent oversampling. The acquired mass range was set between 50 and 1000 *m*/*z*. After acquisition, rhodamine (red ink)-coated slides were used for determining the total impact surface area by spraying the surface for 5 s. The resulting craters on the rhodamine slides were scanned using a Nanozoomer slider scanner (Hamamatsu, Shizuoka, Japan) and its viewing platform NDP Viewer v2.3.1, and the total impact surface area of the desorbed craters was calculated using ImageJ^24−26^ in μm.^2^

### Three-Factorial Experiment for DEFFI

Three parameters were used to conduct a three-factorial experiment for DEFFI, namely, solvent flow rate in μL/mL, voltage in kV, and gas pressure in bar. The remaining parameters were set at 150 μm orifice diameter, 20 μm I.D./360 μm O.D capillary TaperTip, ∼100 μm distance between emitter and orifice, and solvent composition of 95:5 methanol–water, and the metal cap was grounded to earth. The following parameters were used in the three-factorial experiment: (1) solvent flow rate Q_L_: 0.75, 1.5, 3, and 4.5 μL/mL; (2) voltage: 0, 1.5, 3, and 4.5 kV; (3) gas pressure *P*_g_: 1, 3, 5, and 7 bar (Figure S2). Acquisition was performed on 5 lines of 5 mm in length on pork liver sections with 1 scan/sec at an acquisition speed of 100 μm/ms and spacing between row lines of 500 μm, to prevent oversampling. The acquired mass range was set between 50 and 1000 *m*/*z*. After acquisition, rhodamine slides were used for determining the area of total impact surface area by spraying the surface for 5 s. The resulting craters on the rhodamine slides were scanned using Nanozoomer slide scanner as described in the previous section, and the total impact surface area of the desorbed crater was calculated using ImageJ^24−26^ in μm^2^.

### MS Imaging

Imaging was performed using a commercially available DESI source manufactured by Waters, with the optimized parameters according to Tillner et al. (2017).^27^ The DESI sprayer held a TaperTip capillary with an inner diameter of 20 μm and an outer diameter of 363 μm and was operated at an incidence angle of 75° with respect to the plane of the surface, a sprayer-to-inlet capillary distance of 5 mm, and a sprayer-to-surface distance of 1 mm. A high voltage of 4.5 kV was applied. A solvent methanol/water, 95:5 (v/v), delivered by a nanoAcquity binary solvent manager (Waters, Wilmslow, UK) at a solvent flow rate of 1.5 μL/min and an inlet gas pressure of 5 bar (nitrogen) was used. Because the DEFFI sprayer was based on the same commercially available Waters’s DESI sprayer, the geometrical setup remained unchanged. Following the thorough optimization studies, the final conditions used for tissue imaging with DEFFI were as followed: the sprayer held a TaperTip capillary with an inner diameter of 20 μm and an outer diameter of 363 μm, a capillary-to-orifice distance of 100 μm, and an exit orifice diameter of 150 μm. The solvent flow rate was set at 0.75–1.5 μL/min methanol/water, 95:5 (v/v), with an inlet gas pressure of 5 bar (nitrogen) and a high voltage of 4.5 kV.

### Data Processing and Analysis

Waters raw data files were converted into mzXML files using the ProteoWizard msConvertGUI (Vanderbilt University, Nashville, USA). The mzXML file was imported into MatLab (R2014a; MathWorks, Natick, USA) environment using an in-house written function. Two metabolites, 279.23 and 885.55 *m*/*z*, were peak picked, which corresponded to FA(18:2) and PI(20:4/18:0), respectively. The intensity for each of the metabolites was averaged across a single line (5 mm) of acquisition. The mean intensity for each of the metabolites was calculated across 5 lines, with its corresponding standard deviation. Annotation was performed using the Lipid Maps database (http://www.lipidmaps.org/) with an *m*/*z* tolerance of 5 ppm for accurate mass data (Figure S3). The presence and correct abundance of the ^13^C isotope peak was also verified. Optical images of the stained slides were examined and measured using Hamamatsu Nanozoomer, and its NPD Viewer v2.3.1. Ion images were generated using high definition imaging platform version 1.4 (Waters, Wilmslow, UK).

## Results and Discussion

### Optimization of Flow Focusing Parameters

**Figure 2:**
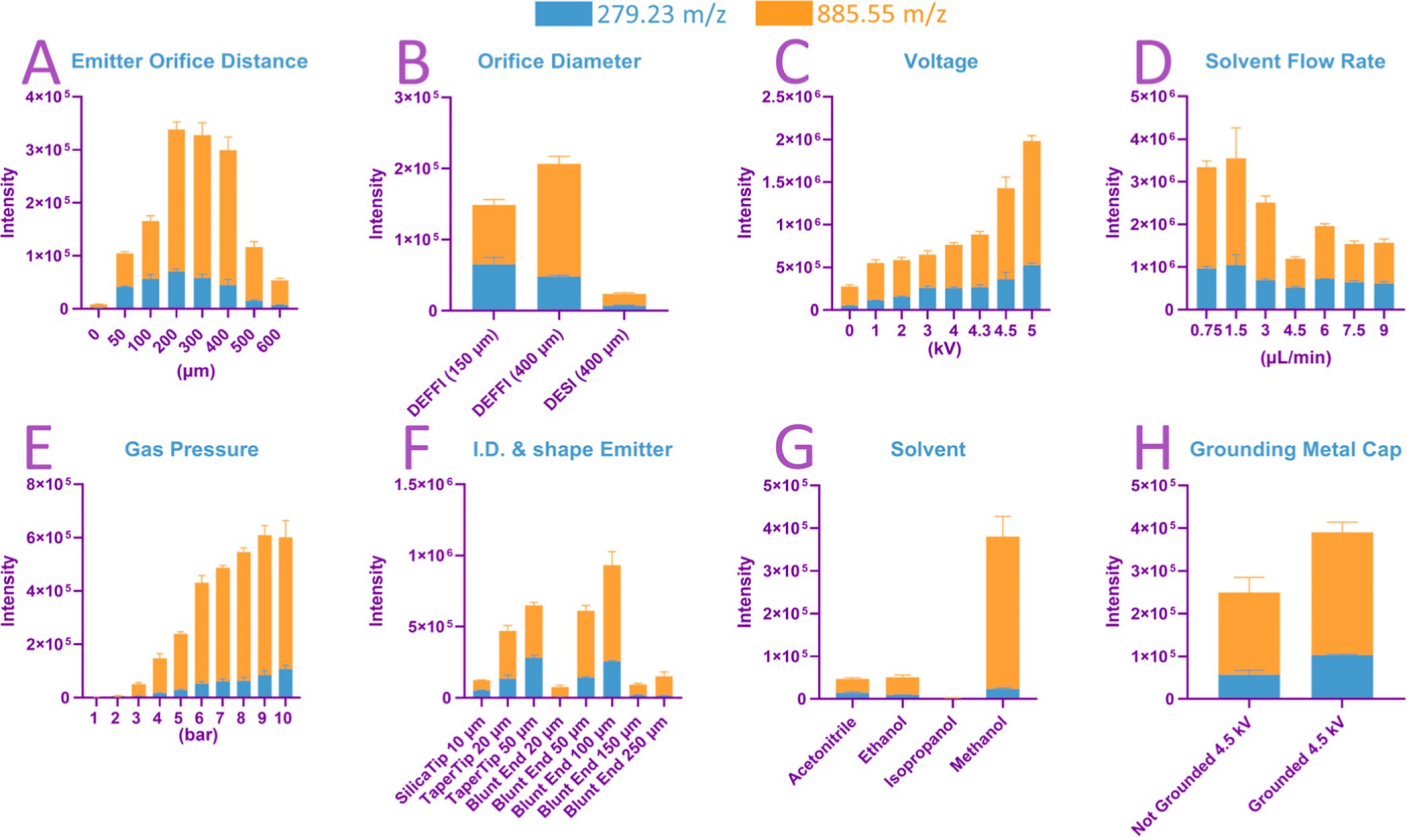
Averaged sum intensity for each parameter. Each bar is shown as sum intensity of fatty acid FA(18:2) *m*/*z* 279.23 and phosphatidylinositol PI(20:4/18:0) *m*/*z* 885.55, under each condition. The following parameters were tested: distance between emitter and orifice in μm; orifice diameter in μm; voltage in kV; solvent flow rate in μL/min; gas pressure in bar; shape of the distal tip and inner diameter of emitter in μm; solvent; and grounding of metal cap.

**Figure 3:**
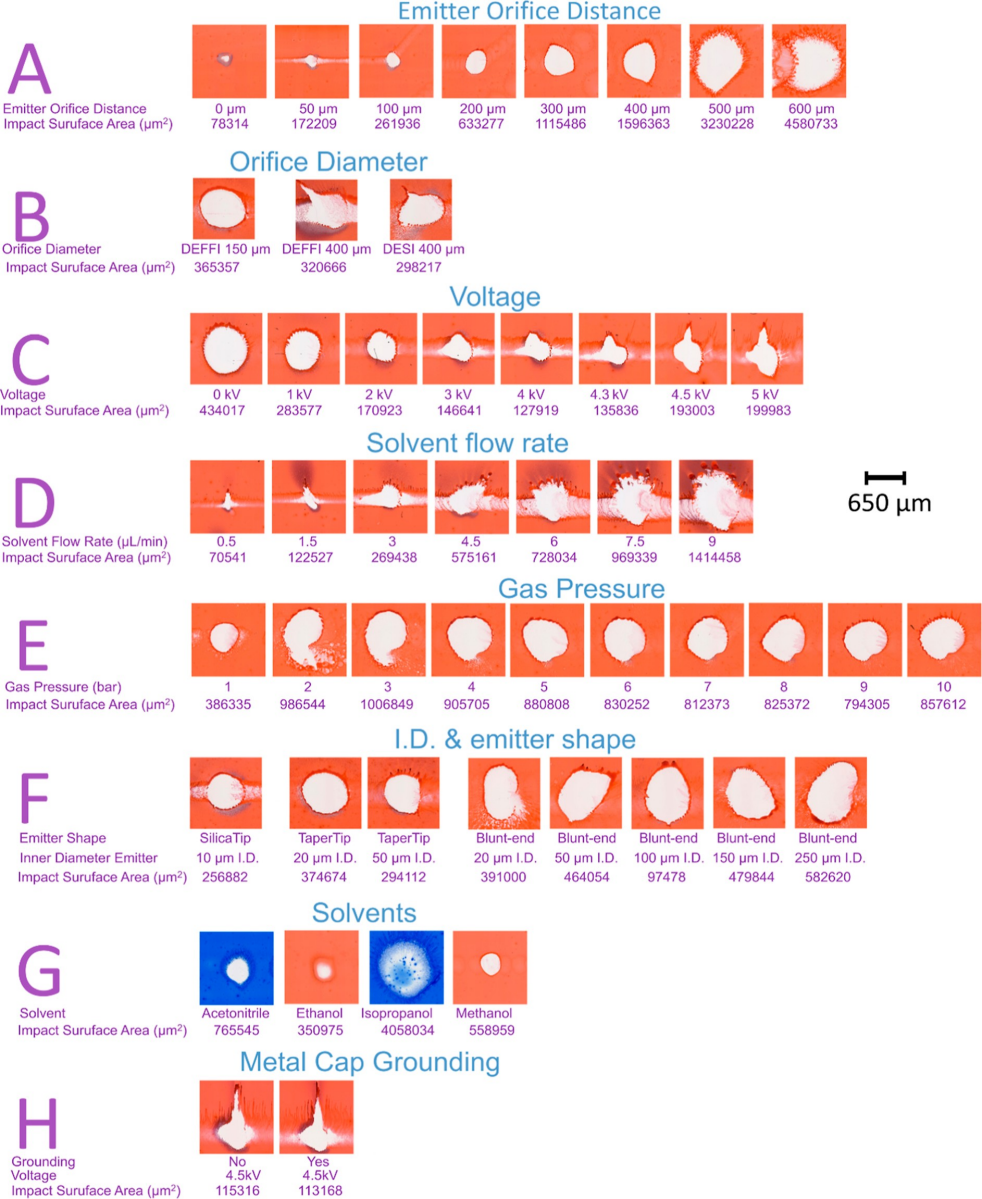
Visualization of desorption crater on a red ink slide. Each spot was sprayed for 5 s for the following tested parameters: distance between emitter and orifice in μm; orifice diameter in μm; voltage in kV; solvent flow rate in μL/min; gas pressure in bar; shape of the distal tip and inner diameter of emitter in μm; solvent; and grounding of metal cap. Total impact surface area of the empty craters was calculated and is shown in μm^2^.

The results of the tested parameters for signal intensity and desorbed surface are shown in Figures 2 and 3, respectively. Each parameter optimization is labeled in an alphabetical order from A to H on both figures.

(A) Emitter-to-orifice distance: The distance between the emitter and orifice (H) was particularly effective within the 0–200 μm range, in producing good signal intensity while maintaining good spatial resolution. *H* = 0 μm, where the distal tip of the emitter was nearly touching the nozzle of the orifice, produced the smallest spray point. This is ideal for imaging at a higher spatial resolution, at the expense of signal intensity. However, *H* = 50–200 μm distance would suffice as part of a general workflow for MSI, without the risk of damaging the tip of the solvent capillary. Previous research has found that the stability of emitted jet is optimal between *H* ≃ *D*/2 and *H* ≃ *D*, for flow focusing and electro-flow focusing, respectively, to yield a stable jet for a given orifice.^18,28−30^ This is also the case for this experiment as *D* was set at 150 μm, where signal intensity was the highest at *H* = 200 μm. However, the smallest jet diameter was produced by the shortest distance between emitter and orifice. (B) Orifice diameter: In the previous experiment A (emitter-to-orifice distance), we concluded an optimal *H* = 200 μm for *D* fixed at 150 μm. For this experiment, *H* was fixed at 400 μm as outlined in the Experimental Section to optimize the remaining parameters. Two different orifice diameters (*D* = 150 μm and *D* = 400 μm in DEFFI mode) were tested, and the experiment yielded a similar impact surface area for both *D*. However, *D* = 400 μm yielded the highest signal intensity compared with *D* = 150 in μm, which corresponded to *H* ≃ *D* in the previous experiment (emitter-to-orifice distance) as *H* was set at 400 μm as a fixed parameter. For the direct comparison with DESI setup according to Tillner et al.*,*^23^*D* was set at 400 μm to allow both the protrusion and retraction of solvent capillary to operate in both DESI and DEFFI modes, respectively. Spectrum from both DESI and DEFFI ionization techniques generated an overall similar spectral profile, albeit with a difference in signal intensity (Figure S4). The overall signal intensity of DEFFI was an order of magnitude higher compared to DESI, while the impact surface areas remained relatively the same. This is most likely due to the retracted solvent capillary in DEFFI that allows solvent and gas to flow concentrically through the orifice without obstruction, which is rarely the case in DESI^14,23^ (Figure S5). An increase in analyte signals generally correlates with an increase in noise signals. However, the overall noise signals were on average 3X more in DEFFI than in DESI, while the overall analyte signals were improved by an order of magnitude (Figure S6). The increase in signal intensity also translates to a greater depth of information where low abundant peaks in DESI may be below the signal-to-noise ratio threshold, which are now observed with sufficient intensity to map their localization within the tissue and to determine a possible molecular identity based on accurate mass value. (C) Voltage: Using this new sprayer configuration it was possible to obtain acceptable levels of signal from the tissue and a perfectly symmetrical spray point without any voltage applied. Similar results have been reported in DESI without voltage, termed as desorption sonic spray ionization (DeSSI) or easy ambient sonic spray ionization.^31,32^ Conversely, DEFFI applied with voltage produced considerably higher signal intensity. Beside ionizing analytes, the applied voltage produces a potential difference capable of propelling solvent droplets at a higher velocity. Higher voltage also seems to produce a smaller impact surface area, which is advantageous for imaging at higher spatial resolution. Gañán-Calvo et al.^33^ reported that increasing the voltage correlates with a decrease of droplet size at 1.25 μL/min, whereas a solvent flow rate at 12.5 μL/min was not affected by the increase of voltage. Similar results were observed in this experiment, where high voltage permitted the formation of very small charged droplets, thereby decreasing the radial dispersion of the solvent jet. Moreover, there seems to be an optimal voltage threshold above which the primary solvent droplets start to become unstable, resulting in a larger total impact surface area as well as causing an asymmetrical spray point. The threshold was found to be above 4.5 kV, and it could be caused by space charge effects. (D) Solvent flow rate: There is a correlation between higher solvent flow rate (*Q*_L_) and bigger impact surface area. The increase in impact surface area could be attributed to larger primarily solvent droplets and an increase in radial dispersion. In addition, higher *Q*_L_ is usually associated with higher signal intensity, which was not the case in this experiment, where the strongest response was observed at *Q*_L_ = 1.5 μL/min. This could be due to the accumulation of larger solvent droplets on sample surface to form a thick liquid film. As a result, the thick liquid film could negatively affect the formation of secondary solvent droplets. A *Q*_L_ between 0.5 and 1.5 μL/min can be used to achieve better spatial resolution. (E) Gas pressure: it was shown previously that good quality spectra can be acquired using a low gas pressure (*P*_g_) between 10 and 20 psi in DEFFI, which is equivalent to 0.7 and 1.5 bar, respectively.^15,22,34^ In our experiment, the sum intensity of the two metabolites correlated with higher *P*_g_, from 1 to 10 bar, while the impact surface area remained relatively the same. While *P*_g_ = 10 bar was possible, *P*_g_ = 5 bar was chosen for the remainder of the experiment, due to safety reasons. (F) Inner diameter of the emitter and the shape of the distal tip of the emitter: blunt fused silica capillaries were used for inner diameter (*D*_1_) comparison (20–250 μm) and an outer diameter of 363 μm. Varying the *D*_1_ of the blunt tip capillaries did not have an effect on the impact surface area, but the highest sensitivity was achieved at *D*_1_ = 100 μm. This trend is also shown when using an emitter with a tapered tip, with an increase from *D*_1_ = 10 μm SilicaTip to *D*_1_ = 20 μm TaperTip, then to *D*_1_ = 50 μm TaperTip, which yielded the highest signal intensity. When comparing the shape of the distal tip of the emitter, there seems to be a pattern that a longer taper tip of emitter produces a slightly smaller impact surface area as well as a more symmetrical spray point. This may suggest that using a longer and sharper edge at the distal tip of capillary can prevent the solvent fluid adhering to the distal edge of the capillary. (G) Solvent: Methanol yielded the highest sensitivity and smallest impact surface area. This is also true for DESI, where methanol has generally been used as the main solvent. This is in line with solvent composition studies done in DESI to improve spectral quality and spatial resolution.^35^ Difference in solvent composition may have an influence on the overall detected signals due to differences in analyte solubility (Figure S7). Methanol has yielded a better sensitivity on phospholipid mass range (600 *m*/*z*–900 *m*/*z*), particularly 885.55 *m*/*z* PI(20:4/18:0), which was a phosphatidylinositol. Phosphatidylinositol is one of the most hydrophilic analytes, so it was expected to be the highest peak using methanol. Conversely, IPA yielded an aberrant spray point and very low signal intensity, as if it was splashing droplets on the surface. This could be due to the high viscosity of IPA that caused high back pressure on the solvent pump and could not provide stable solvent flow. (H) Grounded and non-grounded orifice plate: Grounding the isolated metal cap to earth considerably improved the overall sensitivity. The metal cap with the orifice was isolated from the rest of the sprayer’s conductive components. The strong electric field inside the self-contained sprayer could have exerted a force on the electrically charged solvent droplets, which therefore assisted the ejected velocity of charged solvent droplets through the orifice. The grounding of metal cap may have exerted a bigger force on charged solvent droplets than the source of the mass spectrometry. This also reduced the space charge effects, thereby decreasing the radial dispersion of the ejected solvents to concentrically focus the charged droplets into a more confined space, at a higher velocity. Thus, grounding the nozzle fixes the potential drop from the end of solvent capillary to the orifice, thus reducing the charging region to the nozzle only. Without this the nozzle will be prone to charging up and periodically discharging.

### Three-Factorial Experiment

**Figure 4:**
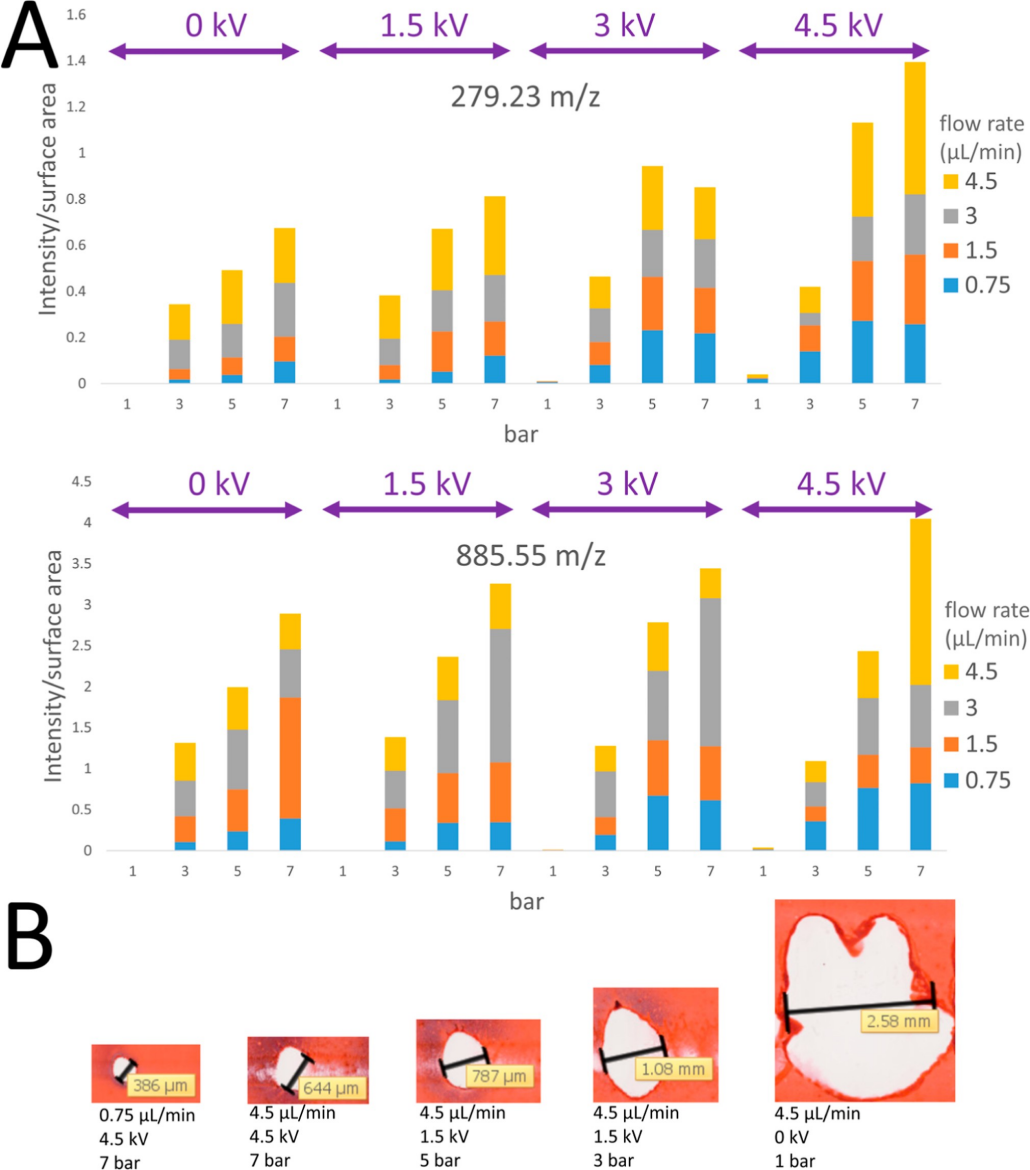
Results from three-factorial experiment. (A) Stacked bar plot displaying the intensity of fatty acid FA (18:2) *m*/*z* 279.23 and phosphatidylinositol PI (20:4/18:0) *m*/*z* 885.55 for each set of parameters (*Q*_L_, kV, and *P*_g_). (B) Visualization of desorbed area on a red ink-coated glass slide: different parameters produce widely different ejected solvent droplets, from big and dispersed to confined eject solvent droplets.

The three main parameters (*Q*_L_, *V*, and *P*_g_) relating to the fluidic properties of the ejected solvent droplets are inter-dependent with regard to their ability to affect the performance of the sprayer. They are also the conditions that are most easily controlled by the user and as such should be investigated in more detail. A three-factorial experiment was conducted to map out a parameter matrix for these three variables to determine how they influenced each other, in terms of signal intensity and impact surface area. The resulting signal intensity and impact surface area varied widely for different combinations of parameters. Thus, the signal intensity was normalized by the total impact surface area, measured as illustrated in Figure 4B, to create a parameter termed desorption efficiency, to obtain the molecular ion yield per unit area^35^ as shown in Figure 4A. Desorption efficiency is directly proportionate with increased *Q*_L_, *V*, and *P*_g_, with the highest desorption efficiency achieved at *Q*_L_ = 4.5 μL/min, 4.5 kV, and *P*_g_ = 7 bar, which would be ideal for screening purposes. However, only *P*_g_ and *V* correlated with improved spatial resolution. Therefore, to achieve a desorption efficiency that yielded a high spatial resolution, it is best to use higher voltage (4.5 kV) and gas pressure (*P*_g_ = 7 bar) and lower solvent flow rate (*Q*_L_ = 0.75 μL/min). It can be observed that these optimal parameters are well outside the standard parameters operated in an electro-flow focusing regime, and as such, the dominant mechanism would be the formation of an electrospray that is subsequently focused by the gas flow through the orifice.

There are noticeable effects of electrification on the impact surface area at different *P*_g_ and *Q*_L_, and these are summarized in Figure S8. The ratio between the impact surface area at applied voltage and at 0 kV was calculated for every condition. It can be observed from this figure that a lower *Q*_L_ was able to produce a significantly smaller impact surface area at higher voltage, whereas a larger *Q*_L_ was less sensitive to voltage in the reduction of impact surface area, which was also observed by Gañán-Calvo et al.^28^ during the measurement of droplet size distribution. In addition, a low *P*_g_ (3 bar) was more sensitive to higher voltage at low Q_L_ (0.75 μL/min) than at high *Q*_L_ (4.5 μL/min) in yielding a smaller impact surface area. This was previously described by Gañán-Calvo et al.^28^ as the electrical field was insufficient to modify the jet surface when residence time (emitter-to-orifice distance divided by linear velocity solvent flow) was smaller than the electrical relaxation time (solvent electrical permittivity divided by solvent conductivity). This means a small dielectric constant and a large conductivity of solvent, small linear velocity of solvent, and large emitter-to-orifice distance are more sensitive to high voltage. As a result, the reduction in impact surface could be attributed to the reduction in radial dispersion of ejected droplets and the reduction of solvent droplet size.

Depending on the viscosity of the liquid and geometry of the flow focusing setup, combinations of varying *P*_g_ and *Q*_L_ are known to affect the properties of the ejected solvent droplets in different regimes. This was evident in the previous section of Optimization of Flow Focusing Parameters and Three-Factorial Experiment, with high voltage as an additional parameter. Closer examination of the DEFFI trace at scan speeds of 30 mm/min on the red ink-coated glass slide showed that the sprayer produced a different spray pattern that did not resemble dripping or jetting (Figure S9). The dripping regime was observed at very low gas pressure and voltage, and it started to transition to jetting regime at slightly higher voltage and pressure, where it started to produce a series of individual droplets. When all the parameters were increased, spots of individual droplets were not distinguishable. The aberrant trace on red ink seemed to indicate turbulence of the ejected droplets. This could indicate either turbulent flow focusing^36^ or flow blurring regime,^37^ as the estimated ratio between *H* and *D* was ∼0.66, which was previously described by Rosell-Llompart and Gañán-Calvo^36^ to be near the boundary of 0.5, which separated capillary flow focusing and turbulent flow focusing with flow blurring. This phenomenon is characterized by a turbulent interaction between liquid and gas phases due to a backflow pattern occurring within the solvent emitter. The intense mixture between liquid and gas phases leads to efficient atomization of solvent droplets. However, this has to be further validated, as a scan speed of 30 mm/min was probably insufficient to resolve the individual droplets on the red ink-coated glass slide.

### Mouse Brain Imaging

**Figure 5:**
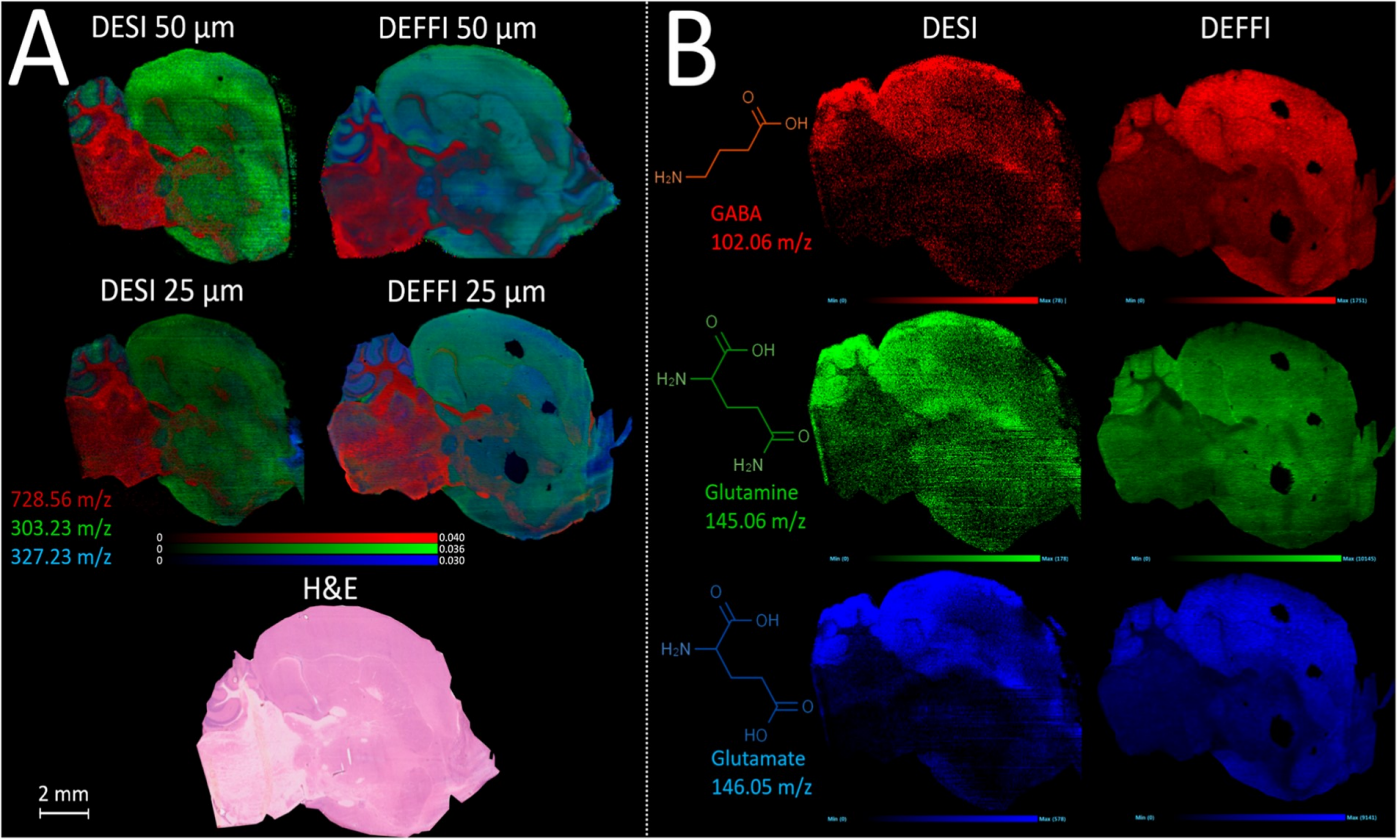
(A) Overlay ion images of phosphatidylethanolamine PE(O-36:2) 728.56 *m*/*z* (red), fatty acid FA(22:6) 327.23 *m*/*z* (blue), and fatty acid FA(20:4) 303.23 *m*/*z* (green), with a pixel resolution of 50μm (top) and 25μm (bottom), compared between DESI (left) and DEFFI (right), on sagittal sections of a mouse brain, acquired in a negative ion mode. (B) Ion image for 3 low-intensity neurotransmitters in mouse brain: GABA 102.06 *m*/*z* (red), glutamine 145.06 *m*/*z* (green), and glutamate 146.05 *m*/*z* (blue), compared between DESI and DEFFI, on a mouse brain with a pixel resolution of 25μm.

Published articles relating to DEFFI have only reported its use for detecting traces of explosives, narcotics, and lotions and on artificial fingerprints from forensic lift tape.^15,22,34^ The present work is the first time that the DEFFI technique was optimized for the imaging of biological samples, with the data presented above obtained from porcine liver section. In order to demonstrate the results of this optimization study, a commonly used and well-understood sample type for imaging mass spectrometry comparisons is presented, in this case mouse brain. Figure 5A demonstrates the results of imaging subsequent sections of the same tissue block by the DESI and DEFFI configurations described above. Both setups were optimized to the best of the operator’s ability and analyzed on the same mass spectrometry instrument. The increased signal intensity observed with the DEFFI configuration as described above is reflected in a greater image quality than the comparative data from the DESI sprayer. Higher peak intensities generally translate to greater contrast in the ion images. This is apparent in the images from DEFFI imaging at 25 μm, which appear sharper to the eye than those from the DESI experiment. Further work will be conducted to calculate the spatial resolution of these systems. However, comparing the 25 μm pixel image data to the 50 μm for DESI and DEFFI, respectively, revealed a greater degree of image sharpness for the DEFFI experiment, whereas the two DESI images are largely similar, suggesting that the sampling cannot match the smaller pixel sizes. Even though the total impact surface area on the red ink slide from the optimization experiment was at best ∼100 μm in diameter, DEFFI was able to produce sharper images acquired at 25 μm pixel resolution, due to the nature of the desorption event. The highest spray density region within the overall impact surface area is generally smaller than its outer region, and it is the most effective area in the desorption and ionization of analytes from the surface.^13^ Therefore, for biological tissue imaging, where a threshold value must be met for effective desorption of secondary droplets from the sample, the region over which this occurs may be smaller than the apparent area covered by the spray on the surface. This is generally not the case for the red ink-covered slide, where the ink being highly soluble in the spray solvent and the glass slide providing no resistance to the formation of secondary droplets means that even the less effective outer plume of the spray can lead to desorption from the sample.

Despite the increased sensitivity of DEFFI when compared to DESI, the tissue integrity remained intact, as H&E staining was performed on the same slide post-acquisition. In addition, the increased sensitivity of DEFFI allowed the structural and spatial visualization for low intensity metabolites such as neurotransmitters γ-aminobutyric acid (GABA), glutamine, and glutamate that were difficult to measure using DESI (see Figure 5B). This was particularly true for GABA using DESI, as shown by the presence of a high number of empty pixels, whereas DEFFI showed the presence of GABA on the entire brain section. This example of the performance enhancement offered by DEFFI offers promise for the application of this approach to a range of biological and medical research, such as the study of the role of neurotransmitters in different types of neurological disorders and molecules that cannot be mapped within tissue easily by any other technology.

## Conclusions

In conclusion, the implementation of a flow focusing mechanism for imaging is highly suitable for biological tissue analysis. Its sensitivity is higher than that of the same sprayer setup run in DESI mode, with the same optimized parameters described by Tillner et al.,^23^ with the only difference being the retracted and protruding emitter from the 400 μm aperture nozzle, for DEFFI and DESI, respectively. Based on known parameters for DESI and DEFFI, the sensitivity and spatial resolution of DEFFI was improved through a systematic optimization study specifically targeted to biological tissue as a sample type.

The retraction of the emitter inside the sprayer has several potential advantages over the protruding emitter found in DESI. The sprayer is rotationally symmetric, which makes it easier to optimize other parameters and potentially reduces variability. The sprayer itself is more robust due to the delicate solvent emitter being retracted inside the sprayer, protecting the tip of the emitter from being damaged. Having a fixed physical geometry allows for easy optimization of the operating parameters, namely, the solvent flow, gas pressure, and voltage. It has been shown here that the best desorption efficiency in DEFFI was achieved at high *V*, *P*_g_, and *Q*_L_, whereas high spatial resolution for imaging purposes was achieved at high *V* and *P*_g_ and low *Q*_L_. In addition, good-quality spectra and images can still be acquired in the absence of applied voltage, albeit slightly lower spatial resolution, making it a pure flow focusing sprayer. This form of screening or imaging in the absence of voltage could be a better match for portable instruments and clinical applications if voltage is of concern. Overall, the implementation of the flow focusing mechanism in desorption ionization, by retracting the solvent capillary so that it is positioned behind the orifice of the gas cone, has shown great promise to overcome the current limitations experienced with DESI and can potentially be seamlessly integrated into existing DESI workflows.

## Supporting Information Available

Additional information and Figures as described in the text (PDF)
