# Supplementary material for: High Resolution Ambient MS Imaging of Biological Samples by Desorption Electro-Flow Focussing Ionization
Source: Anal Chem. 2022 Jul 5;94(28):10035–44. doi: 10.1021/acs.analchem.2c00345 (PMC9310024; doi:10.1021/acs.analchem.2c00345)
Supplement: Supplementary file 1 — ac2c00345_si_001.pdf [file ac2c00345_si_001.pdf]

Supplementary Information

For

High Resolution Ambient MS Imaging of Biological Samples by Desorption Electro-Flow Focussing Ionization

Vincen Wu[a], Jocelyn Tillner[a,c], Emrys Jones[d], James S. McKenzie[a], Dipa Gurung[a], Anna Mroz[a], Liam Poynter[b], Daniel Simon[a], Cristina Grau[f], Xavier Altafaj[g], Marc-Emmanuel Dumas[a], Ian Gilmore [c], Josephine Bunch[c,e], Zoltan Takats[a,e]

[a] *Department of Digestion, Metabolism and Reproduction, Sir Alexander Fleming Building, Imperial College London, London SW7 2AZ, United Kingdom*

[b] *Department of Surgery & Cancer, Metabolism and Reproduction, Sir Alexander Fleming Building, Imperial College London, London SW7 2AZ, Unite Kingdom*

[c] *NiCE-MSI, National Physical Laboratory (NPL), Hampton Road, Teddington, Middlesex, TW11 0LW, United Kingdom*

[d] *Waters Corporation, Altrincham Road, Wilmslow, SK9 4AX, United Kingdom*

[e] *Biological Mass Spectrometry, Rosalind Franklin Institute, Harwell Campus, Didcot, OX11 0QS, United Kingdom*

[f] *Neurometabolic Unit, Department of Neurology, Hospital Sant Joan de Déu, 08950 Barcelona, Spain*

[g] *Neurophysiology Laboratory, Department of Biomedicine, Faculty of Medicine and Health Sciences, Institute of Neurosciences, University of Barcelona, 08036 Barcelona, Spain*

Corresponding Authors:

*Zoltan Takats, z.takats@imperial.ac.uk

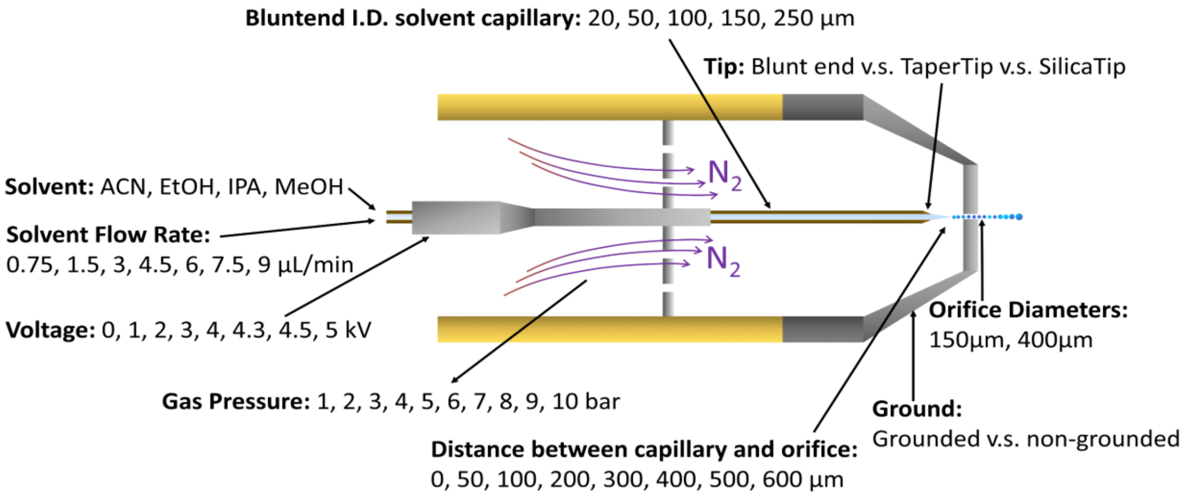

Figure S1. A schematic design of the flow focusing design of the DESI sprayer illustrating the different parameters to be optimized. (1) the shape of the distal tip of the emitter: SilicaTipTM (New Objective, Woburn, MA, USA), TaperTip^{TM} (New Objective, Woburn, MA, USA), and blunt capillaries; (2) inner diameter of emitter (20, 50, 100, 150, and 250 µm); (3) solvents (ACN, EtOH, IPA, MeOH); (4) gas pressures (1-10 bar); (5) distance between emitter and orifice (0, 50,100, 200, 300, 400, 500, and 600 µm); (6) orifice diameter (50, 150, 200, and 400 µm); (7) voltage (0, 1.5, 3, 4, 4.3, 4.5, and 5 kV); (8) solvent flow rate (0.75, 1.5, 3, 4.5, 6, 7.5, 9 µL/min); (9) Grounded and non-grounded metal cap.

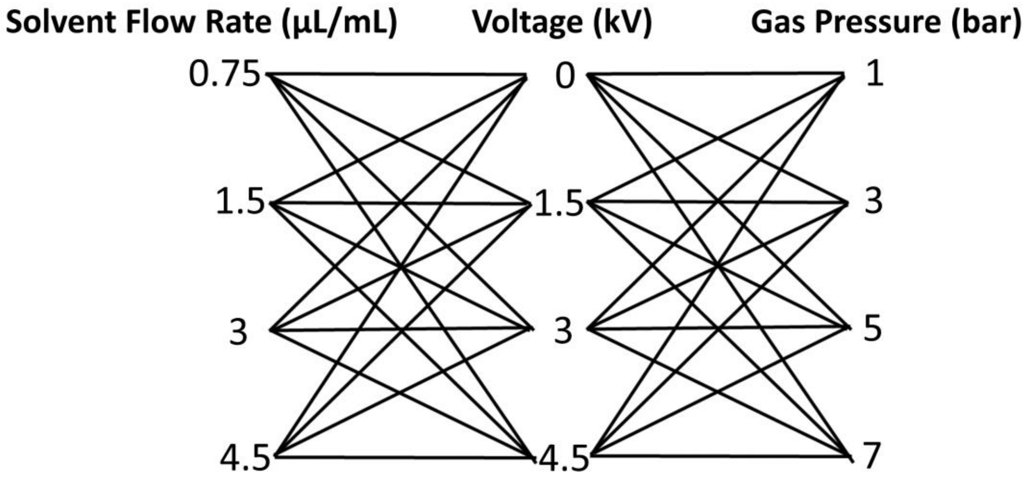

Figure S2. Three Factorial design. Different solvent flow rate, voltage, and gas pressure were tested in a 3 factorial experiment to assess signal intensity and spatial resolution, between regime between dripping and jetting. (1) solvent flow rate 0.75, 1.5, 3, 4.5 µL/mL; (2) voltage: 0, 1.5, 3, 4.5 kV; (3) gas pressure: 1, 3, 5, 7 bar.

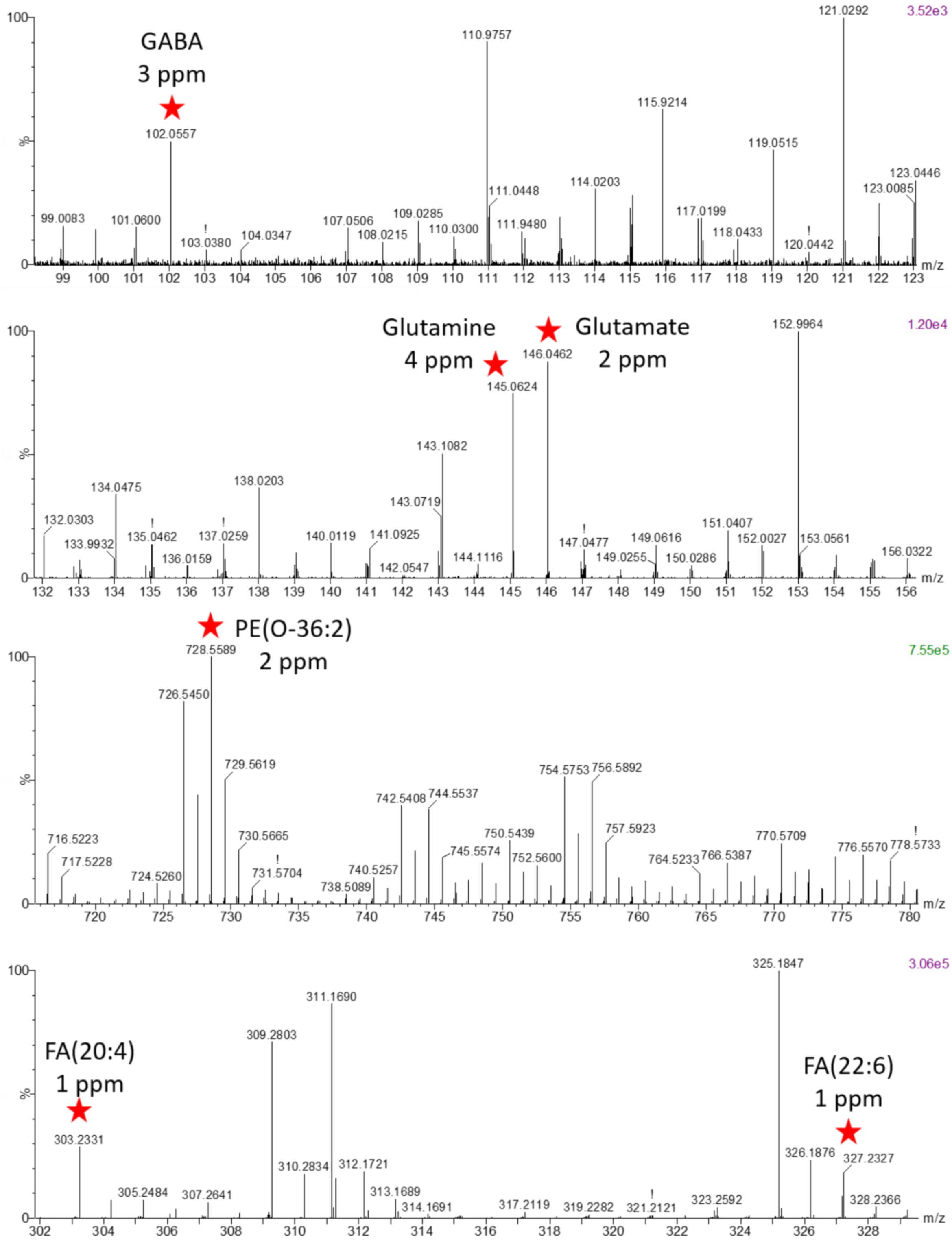

Figure S3. Analytes annotation based on accurate mass from mouse brain extracted spectrum. GABA 3 ppm: 102.0557 m/z, glutamine 4 ppm: 145.0624 m/z, glutamate 2 ppm: 146.0462, PE(O-36:2) 2 ppm: 728.5589, FA(20:4) 1 ppm: 303.2331, FA(22:6) 1 ppm: 327.2327.

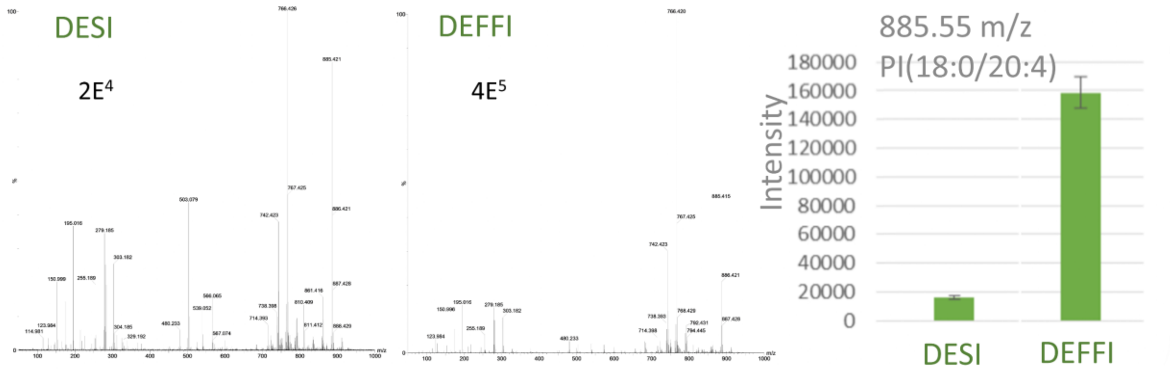

Figure S4. An overview of a single pixel spectrum extracted from pork liver. Comparison of spectra between DESI and DEFFI, using exactly the same sprayer body, geometrical and parameter setup. The only difference between the two sprayers is the solvent capillary being protruded in DESI and retracted in DEFFI.

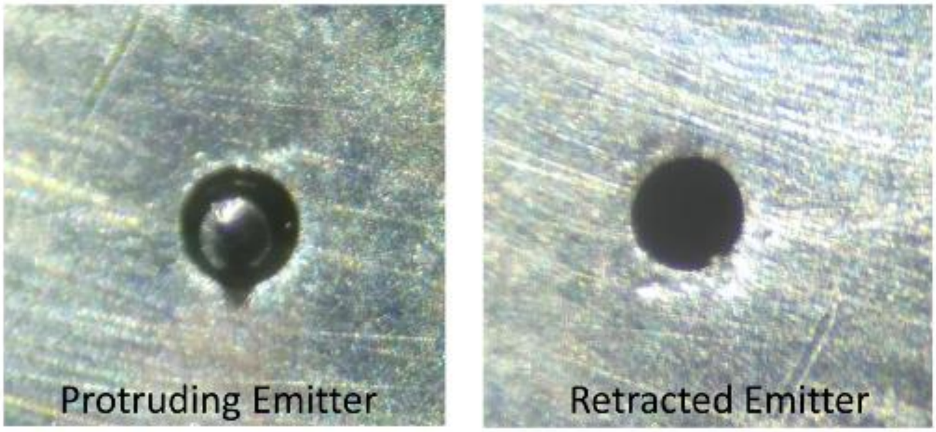

Figure S5. Optical zoom of orifice of metal cap nozzle. (Left) is the protruding capillary out of the nozzle, where it is laid against the side of the orifice. (Right) is the retracted capillary inside the sprayer.

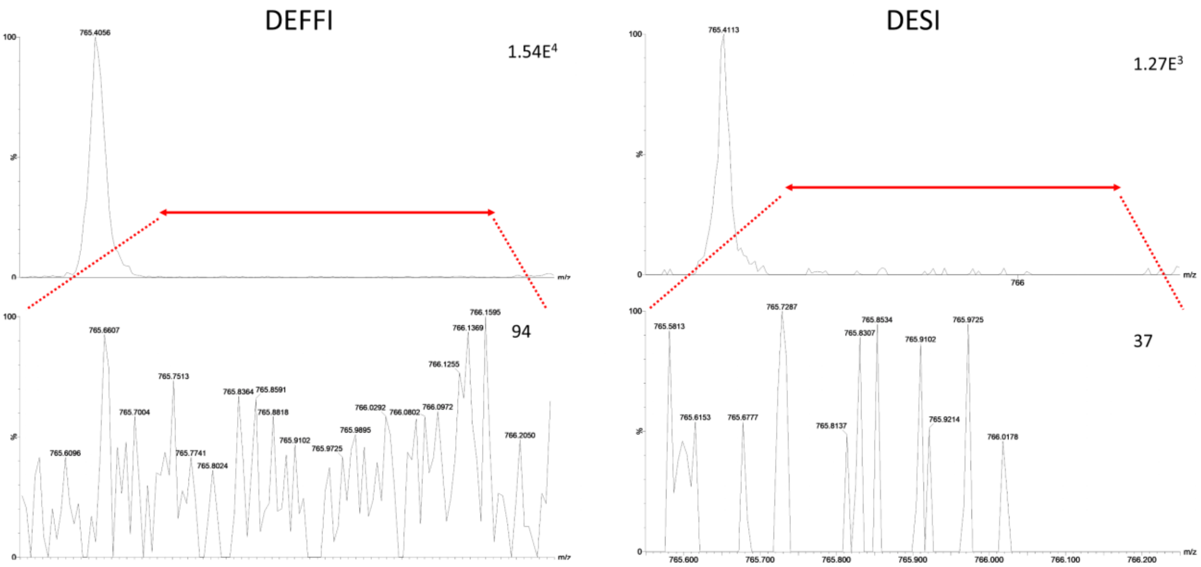

Figure S6. Signal to noise comparison between DESI (Left) and DEFFI (Right). Close-up inspection on the extracted spectrum for an analyte peak and its surrounding noise signals.

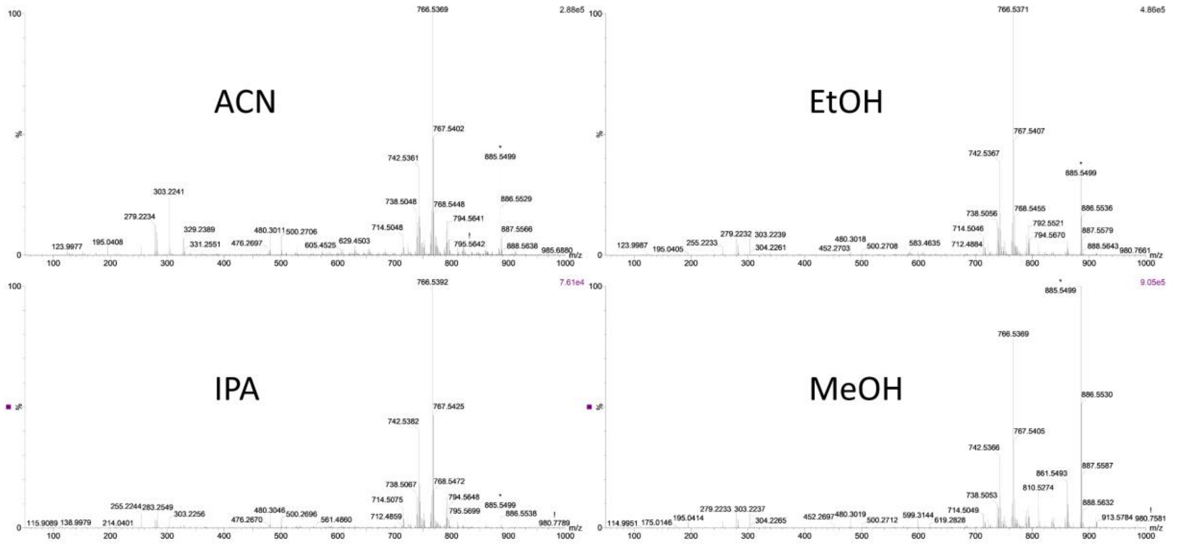

Figure S7. Solvent spectra comparison. The overall metabolic coverage (50-1000 m/z) of pork liver in negative mode between acetonitrile, ethanol, isopropanol and methanol.

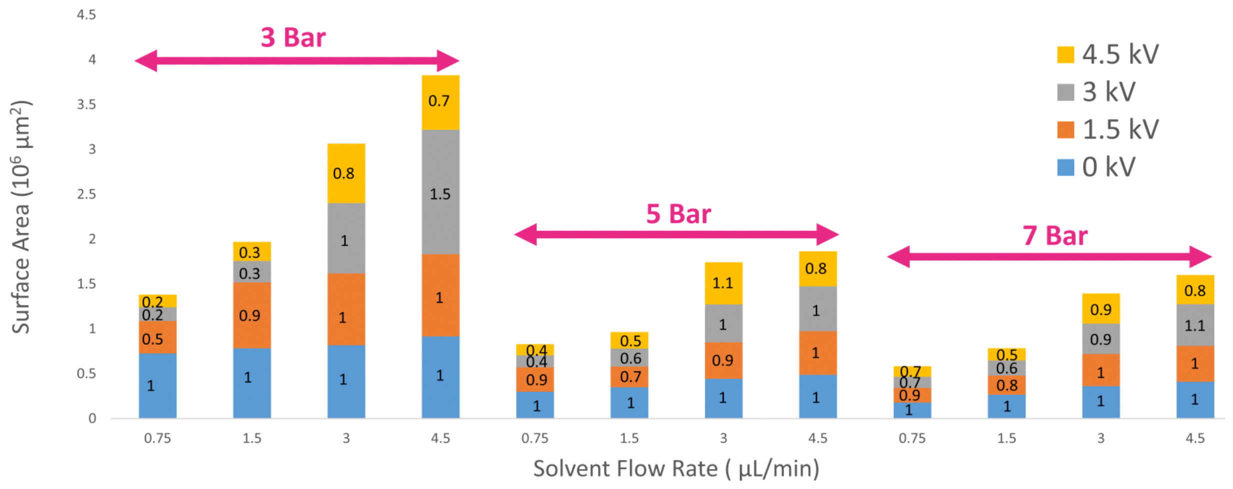

Figure S8. Effect of solvent flow rate, voltage and gas pressure on impact surface area. Effect of different voltage (0, 15, 3, and 4.5 kV) on the total impact surface in µm^2, at different gas pressure (3, 5, and 7 bar), and different solvent flow rate (0.75, 1.5, 3, 4.5 µL/min). The numbers displayed on each stacked bar was the calculated ratio of impact surface area between applied voltage and 0 kV for every condition.

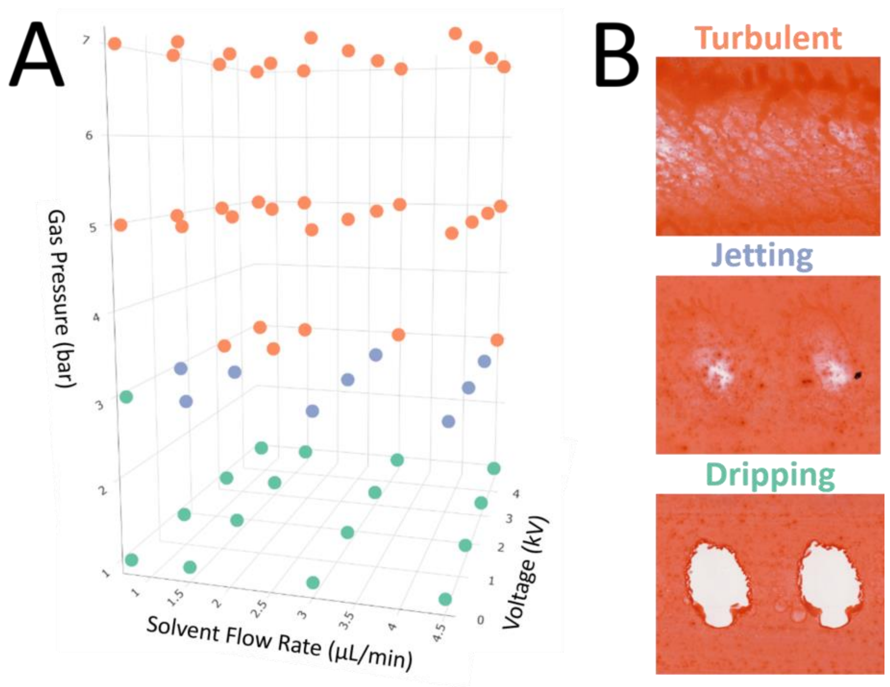

Figure S9. Dripping, jetting, and turbulent flow regime. (A) The influence of gas pressure, voltage, and solvent flow rate on the properties of the ejected solvents droplets. (B) Desorption traces on red ink slide after being rastered with the sprayer at very fast speed at 30000 µm/min (images not to scale).
